# Integrating genomics and transcriptomics reveals candidate genes affecting loin muscle area in Huaxi cattle

**DOI:** 10.1371/journal.pone.0322026

**Published:** 2025-05-09

**Authors:** Qingqing Xue, Lili Du, Tianyu Deng, Mang Liang, Keanning Li, Li Qian, Shiyuan Qiu, Yan Chen, Xue Gao, Lingyang Xu, Zezhao Wang, Caihong Zheng, Lupei Zhang, Junya Li, Huijiang Gao

**Affiliations:** 1 Department of College of Animal Science and Veterinary Medicine, Heilongjiang Bayi Agricultural University, Daqing, Heilongjiang, China; 2 Department of Institute of Animal Science, Chinese Academy of Agricultural Sciences, Beijing, China; South China Agricultural University, CHINA

## Abstract

Loin muscle area (LMA) is an indicator of carcass composition and is related to weight gain, animal musculature and meat quality traits. Therefore, integrating multi-omics data to reveal candidate genes affecting LMA has attracted extensive attention. We used the combined analysis method of GWAS and RNA-seq to find the candidate genes that affect the size of LMA. The association of 770K SNPs with the LMA captured four significant SNPs within or near three genes. Additionally, seven overlapping genes regarding LMA were determined via the analysis of differentially expressed genes (DEGs) and weighted gene co-expression network analysis (WGCNA). There is an overlapping gene (*CD93*) between the results of GWAS and DEGs. Through functional enrichment analysis of the above genes, candidate genes were identified as *THBD*, *CD93*, *RIMS2, PLP1*, *SNCA*, and *NDUFS8*, and it was found that they mainly affected the size of LMA by affecting muscle fiber diameter, muscle cell development, differentiation, and function. The findings provide valuable molecular insights into the mechanisms that influence LMA content in beef cattle.

## Introduction

Beef is a source of high-quality protein, beneficial fatty acids, and various trace elements in the human diet, which plays an essential role in maintaining the healthy diet [1]. Improving meat quality traits in beef cattle is a key issue facing the beef cattle breeding industry and a hot research topic for beef cattle breeding scholars. Loin muscle area (LMA) is the surface area of the longissimus dorsi muscle (ribeye) at the 12th to 13th rib interface of beef cattle. Previous studies have estimated the heritability of LMA to range from 0.24 to 0.32 [2–4], indicating that it is a moderately heritable trait that can be improved through selective breeding. The identification of genes that influence LMA in beef cattle is important, especially in the improvement of meat quality and breeding. LMA is closely related to net meat yield and slaughter rate, implying that net meat yield and slaughter rate can be increased by increasing the LMA, which is essential for improving the economic value of beef cattle.

Huaxi cattle, is a new specialized beef cattle breed in China, which consists of 24/32 Northern America beef Simmental cattle, 5/32 of dual-purpose Simmental cattle from Germany and Austria, 1/32 Charolais cattle, 1/32 Sanhe cattle, and 1/32 Mongolian cattleSince the beginning of breeding in 1978, Huaxi cattle have shown good performance in growth rate, slaughter rate and net meat rate [5].

Genome-wide association analysis (GWAS) refers to the analysis of the association between the molecular marker data obtained from the scan and phenotypic traits through genome-wide scanning, to identify genes that affect certain phenotypic or quantitative traits. As an effective molecular genetics method, GWAS plays an important role in the identification of functional genes for quantitative traits and the study of genetic variation of important economic traits in livestock and poultry [6]. A series of genetic loci significantly associated with beef carcass traits have been identified using GWAS. For example, *CARTPT* gene affects LMA by encoding multiple peptides that affect tissue growth and development [7]. The *CHKA* gene [8] influences backfat thickness (BFT) by influencing lipid biosynthesis.

RNA sequencing (RNA-seq) enables the study of gene function and regulatory mechanisms at the global level and the discovery of candidate genes associated with specific functions [9]. The analysis of differentially expressed genes (DEGs) and weighted gene co-expression network analysis (WGCNA) were two mainstream strategies for transcriptomics analysis [10]. The analysis of DEGs is a basic method to compare the differences and similarities of gene expression between two groups of different samples, which can obtain the genes that are significantly up-regulated and down-regulated in one group of samples compared with the other group. Further study of the function of these DEGs will eventually detect the vital genes that affect the target traits. The WGCNA approach aims to find co-expressed gene modules and to explore the associative relationship between the gene network and the phenotype of interest, as well as the core genes in the network. Santos Silva used RNA-seq technology to identify DEGs in the longissimus dorsi muscle of Neolore cattle and predicted that three key genes were related to LMA [11]. Wang H [12] used the WGCNA method to detect six gene modules significantly related to sex between obese boars and sows and finally identified seven DEGs affecting sex. Yang M [13] identified the *DBI* gene as a candidate gene affecting intramuscular fat in Beijing black pigs using a combined analysis of GWAS and RNA-seq. The *FGL1* gene had a significant effect on LMA [14]. GWAS was performed on Simmental beef quality traits to discover 19 candidate SNP loci and 11 candidate genes associated with five meat quality traits such as meat color and LMA, among which *KCTD16* and *LOC506594* were related to LMA [15]. Although numerous studies on LMA have performed, DEGs affecting LMA in Huaxi cattle still need to be further determined. Therefore, this study aimed to identify candidate genes affecting the size of LMA in Huaxi cattle using the combined analysis of GWAS and RNA-seq.

## Materials and methods

### Institutional review board statement

The animal study protocol was approved by the Animal Welfare and Ethics Committee of Institute of Animal Science, Chinese Academy of Agricultural Sciences (IAS2020–21; Beijing, China).

### Samples collection and measurement

The 1,507 experimental individuals in this study were from the Chinese Huaxi cattle resource group, which was established by the Bovine Genetic Breeding Team of the Institute of Animal Sciences of the Chinese Academy of Agricultural Sciences. Blood samples were collected for genotyping during routine farm quarantine procedures. All experimental animals were slaughtered in strict accordance with GB/T 27642–2011. Cattle to be slaughtered are electrocuted and then bled for slaughter. At the time of slaughter, Samples of the left longissimus dorsi muscle from all male individuals were collected and placed into 5 mL freezing tubes, which were immediately frozen in liquid nitrogen and subsequently transferred to a liquid nitrogen tank for long-term storage. These samples will be used for transcriptome sequencing. The section of 12 rib Loin muscle of Huaxi cattle was drawn with sulfuric acid paper [11,16]. Subsequently, the drawn LMA is placed on the measuring board, the number of dots in the measuring board is counted, and the LMA is calculated according to the number of dots, where one dot represents an area of 1 cm^2^. Bulls with an average body weight of 665 kg and an average age of 25 months were selected and divided into two groups for differential expression analysis: a high loin muscle area group (HLMA, n = 6) and a low loin muscle area group (LLMA, n = 6). Additionally, WGCNA analysis was conducted on bulls (n = 20) with the same average body weight and age.

### Genotyping and quality control

Genomic DNA was extracted from blood samples using a TIANamp Blood DNA Kit (Tiangen Biotech Company Limited, Beijing, China). DNA with 260/280 ratios between 1.8 and 2.0 was further analyzed and genotyped by 770K SNPs Illumina BovineHD BeadChip (Illumina Inc., San Diego, California, USA).

Using PLINK (v1.90) for individuals and SNPs quality control. The quality control criteria are as follows: SNPs with deletion rates greater than 0.05, individuals with genotype deletion greater than 10% [17], SNPs with minor allele frequency (<0.05) and Hardy-Weinberg balance test (*P* < 1e-6) [18], and individuals with heterozygosity levels higher than 3 standard deviations were excluded [19]. Finally, 1,488 samples and 605,671 SNPs were remained for subsequent analysis.

### Single-trait GWAS analysis

In this study, according to the actual situation of resource groups, the significance test was conducted on the variables influencing the original phenotypic data, and the significant variables were used to adjust the phenotypic values accordingly. The model is as follows [10]:


$$y_{ijk}=\mu +sex_{i}+field_{j}+year_{k}+e_{ijk}$$ (1)

$y_{ijk}$ is the phenotypic value; μ is the population mean; sex, field, andyear as fixed factors;e is the residual after the normal distribution $N\left(0,I\sigma^{2}\right)$, where I is the identity matrix. Through the significance test, sex and year were selected as fixed factors to be incorporated into the linear model, and the phenotypic values were corrected.

Subsequently, PLINK (v1.90) software was used to calculate the principal components to correct the population stratification, and then using the twstats method to test the significance of the principal components. The results showed that the P-values of the first three principal components were less than 0.05, thus the first three principal components were selected to be as covariables.

This study selected the first three principal components to control for population stratification effects. The Fixed and Random Model Circulating Probability Unification (FarmCPU) method was used to evaluate the population structure, as this model offers higher statistical power and computational efficiency compared to general linear models and mixed linear models. The rMVP package (v1.0.5) was used to explore the association between genomic variations and phenotypes [20].

The significance threshold for GWAS was set at *P = *0.05/N, where N is the number of quality-controlled SNPs. SNPs with p-values below this threshold were considered significant. These significant SNPs were then mapped to the bovine reference genome ARS-UCD 1.2, and linked genes within 100Kb upstream and downstream of these SNPs were identified using the BioMart module on the Ensembl website. These genes were further evaluated by combining literature reviews and gene function annotations to determine their potential influence on LMA trait.

### RNA-seq library preparation

Following the manufacturer’s instructions, the TRIzol reagent (Invitrogen, Life Technologies) was used to extract total RNA from LMA of Huaxi The concentration, quality, and integrity of RNA samples were examined by Nano photometer Spectrophotometer (Thermo Fisher Scientific, MA, USA) and RNA Nano 6000 Assay Kit of the Bioanalyzer 2100 system (Agilent Technologies, CA, USA). To ensure suitability for constructing sequencing libraries, all RNA samples had OD260/OD280 values between 1.8 and 2.0, and an RNA integrity number (RIN) greater than 7.0. The qualified samples were then sent to the Illumina NovaSeq 6000 platform for paired-end RNA sequencing (read length 150 bp). The RNA sequencing was completed by Beijing Novogene Technology Co., Ltd.

### RNA-seq data preprocessing

After the sequencing data were obtained, FastQC (v0.11.4) software was used to detect the quality of original Reads (mass fraction, GC content, length distribution, etc.), and Trimmomatic (v0.39) was employed for quality control including removing low-quality sequences and splitter sequences, resulting high-quality clean reads for further analysis. The index was constructed using Hisat2 (v2.2.1) software and clean reads were compared to the bovine reference genome (ARS-UCD 1.2), and then the SAM files were converted to BAM files by SAMtools (v1.11) software. Finally, the raw count file of gene expression was obtained by using FeaturesCounts (v1.5.2) software. The reading numbers of all samples were combined. Per kilobases per million read (FPKM) values were calculated using R.

### Identification of differential gene expression

The high (H) and low (L) LMA groups each consisted of six Huaxi cattle, with one group having high LMA and the other having low LMA. The raw count of individuals in the two groups was analyzed by DESeq2 (v1.18.0) software. The criteria for DEGs were |log2FoldChange| ≥ 1 and FDR < 0.05. Subsequently, the Kyoto Encyclopedia of Genes and Genomes (KEGG) pathway enrichment [21] and Gene Ontology (GO) annotation [22] were performed for these genes using the clusterProfiler R package (v4.12.0) [23]. GO annotation covers three categories: Biological process (BP), Cellular Component (CC), and Molecular function (MF). The significance threshold of enrichment was set as *P* < 0.05.

### Weighted gene co-expression network analysis

Weighted gene co-expression network analysis (WGCNA) is used to identify clusters (modules) of highly correlated genes, summarize these modules using either the module eigengene or an intramodular hub gene, and explore relationships between modules, as well as their associations with external sample traits through eigengene network analysis. Additionally, WGCNA allows for the calculation of module membership measures [24]. This experiment used the WGCNA R package (v1.72) to construct a gene co-expression network of all genes and LMA trait in 20 samples. Hub genes in the significance module were defined with Gene significance (|GS|) was greater than 0.2 and the absolute value of Module membership (|MM|) was greater than 0.8.

### Construction of protein interaction network and further identification of core genes

Constructing protein-protein interaction (PPI) networks for Hub genes within each significant module [25] via the STRING database. Then, the intersection and visualization of the top ten significant Hub genes of each significant module based on Maximum Clique Centrality (MCC), Neighborhood Component Centrality (MNC), and Degree were identified by the CytoHubba plug-in of Cytoscape software [26]. Finally, The selected genes were further intersected with DEGs for additional analysis. KEGG pathway enrichment and GO annotation were subsequently conducted on the KOBAS website for both these genes and the genes identified through GWAS.

## Results

### GWAS results

After quality control of the selected samples, 1,488 samples as well as 605,671 SNPs were subjected to GWAS analysis.

**Fig 1:**
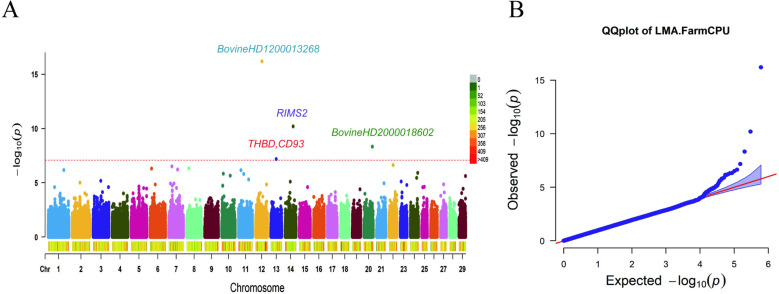
Genome-wide association analysis of LMA trait in the Huaxi cattle population. (A) Manhattan plot. The red dotted lines indicated the significant threshold (*P* = 0.05/605,671). (B) QQ plot.

The heritability of LMA was calculated as 0.43 using GCTA [27], which is high heritability, indicating that the trait can be improved by genetic methods. The Manhattan plot in Fig 1A showed that four significant SNP loci were screened at the genome-wide level (*P* = 0.05/605,671); and the QQ plot in Fig 1B indicated that there was a consistent relationship between the actual and theoretical values for most of the loci, which effectively demonstrated the effectiveness of this method in population control [28]. The basic information of these four significant SNP is shown in Table 1.

**Table 1:** The annotation of significant SNPs.

SNP ID	Chr	POS	REF	ALT	SE	P.adj
BovineHD1200013268	12	48368923	A	G	0.568644443	6.41E-17
BovineHD1300012261	13	42247767	A	G	0.418439684	6.68E-08
BovineHD1400017455	14	62769117	A	C	0.91433653	6.31E-11
BovineHD2000018602	20	65011159	A	G	0.752846917	4.68E-09

Based on the BioMart module, these significant SNPs were identified to be located in or close to three coding genes. BovineHD1300012261 on Bos taurus autosomes (BTA) 13 was located in or close to the *THBD* gene and *CD93* gene; BovineHD1400017455 on BTA 14 was located in the *RIMS2* gene. The two significant SNPs located on chromosome 12 as well as 20 did not detect reveal any associated genes.

### Results of transcriptome sequencing data comparison

S1 Table in S1 File demonstrated the basic statistics for the LMA in the transcriptome 12 experimental Huaxi cattle. After quality control of the transcriptome sequencing data, an average of 21,848,620 clean reads were obtained for each sample. The comparison results showed that the multiple mapping rate, the unique mapping rate, the total mapping rate, and the unmapped rate of the 12 samples were 2.74% ~ 3.79%, 90.08% ~ 91.83%, and 95.97% ~ 97.12%, and 5.16% ~ 6.69%, respectively (S2 Table in S1 File), which indicated that the sequencing results were better to be used for the subsequent analysis.

### Identification of DEGs

**Fig 2:**
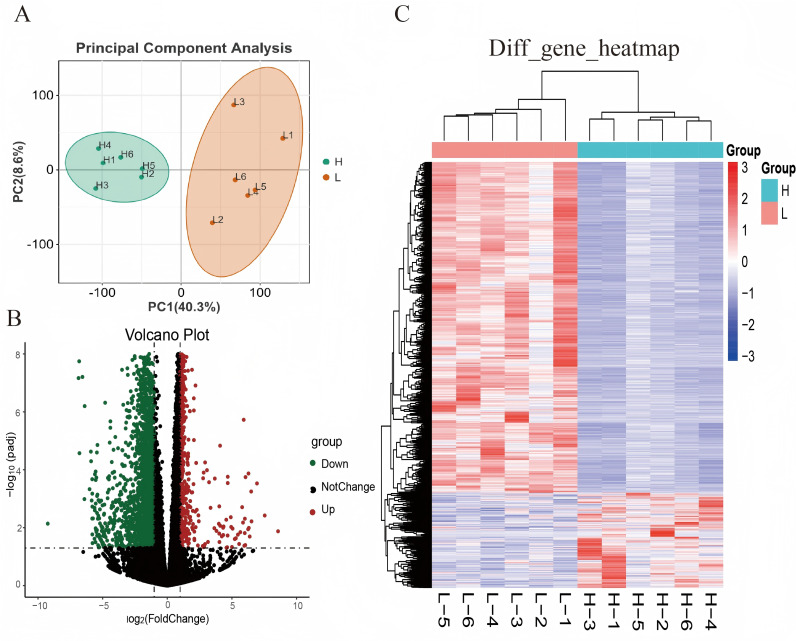
Identification of DEGs in the high LMA and low LMA groups. (A) PCA of the samples. Green and red dots indicated samples with high LMA and low LMA, respectively. (B) Volcano plot of DEGs. Red dots represented significantly up-regulated genes; dark-green dots indicated significantly down-regulated genes; black dots indicated genes with no significant effect. (C) Heatmap of DEGs. H: high LMA group; L: low LMA group.

The individuals in high LMA and low LMA groups were screened according to the values of LMA trait. Principal component analysis was performed on the gene expression data of the two groups of individuals after quality control. Fig 2A showed the sample cluster graph based on the first two principal components. PC1 and PC2 explained 40.3% and 8.6% of the gene expression variation, respectively. DEGs were screened with | log2FoldChange |≥1 and FDR < 0.05. Finally, a total of 2,896 DEGs were identified in the comparison group of HLMA and LLMA, including 647 up-regulated genes and 2,249 down-regulated genes (Fig 2B). Heatmap demonstrated that the expression patterns of these differential genes were similar within groups while different between groups (Fig 2C).

### Functional enrichment analysis of DEGs

**Fig 3:**
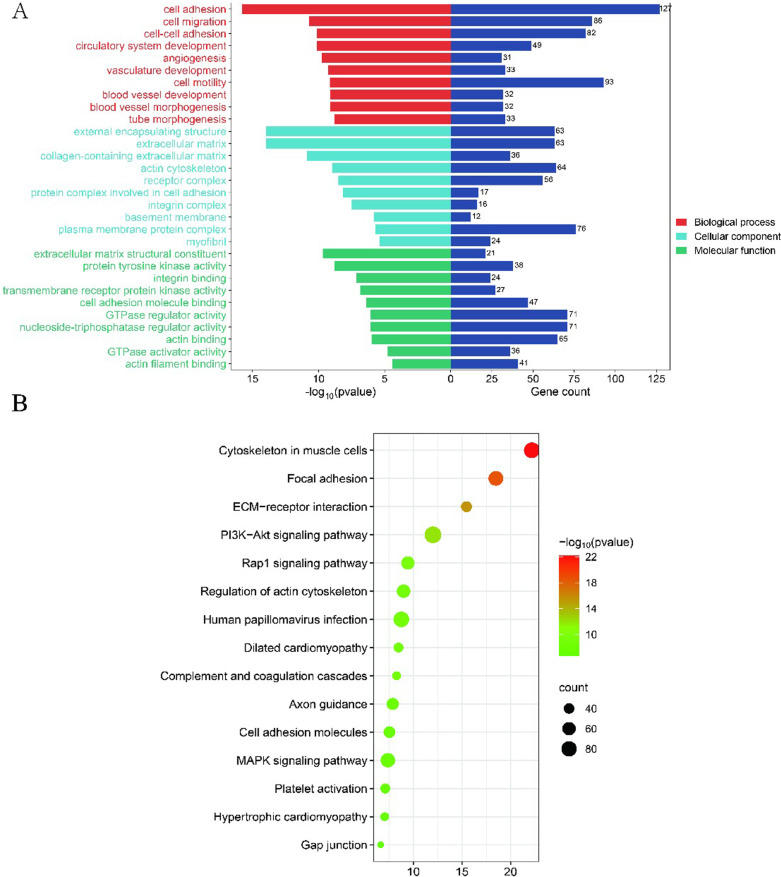
Functional enrichment analysis of DEGs. (A) BP, CC, and MF showed the top ten terms, respectively; (B) The top 15 KEGG pathways enrichment of significant DEGs.

To further understand the biological function of DEGs, 2,896 DEGs were enriched by GO and KEGG. The results showed that DEGs were enriched in 268 GO terms, including 203 biological processes (BP), 36 cellular components (CC), and 29 molecular functions (MF). These DEGs were mainly concentrated in cell adhesion (GO:0007155), actin cytoskeleton organization (GO:0030036), and muscle cell differentiation (GO:0042692) (Fig 3A). Furthermore, these DEGs were enriched in 103 KEGG pathways (Fig 3B), among which the pathways related to muscle development included MAPK signaling pathway (bta04010), Wnt signaling pathway (bta04310), and mTOR signaling pathway (bta04150) could be served as important pathways affecting LMA trait.

### Construction of weighted gene co-expression network

**Fig 4:**
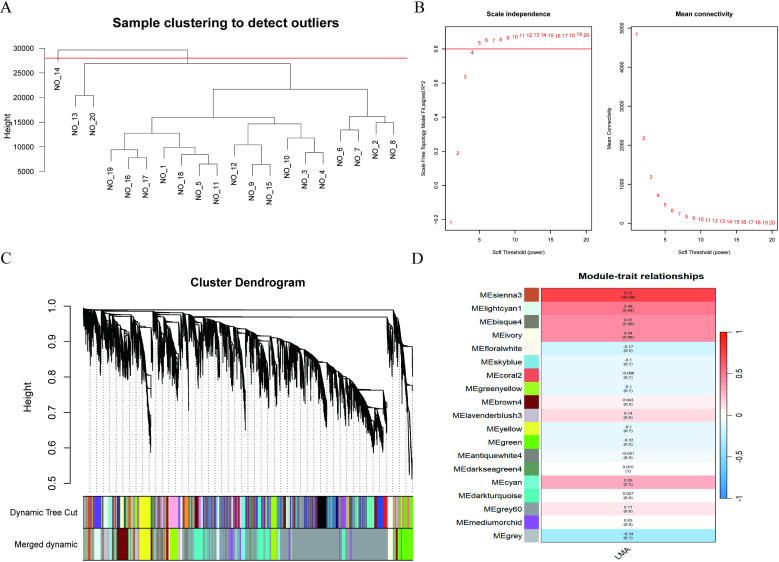
WGCNA analysis. (A) Sample clustering tree. (B) Soft threshold estimation based on adjacency matrix. (C) Dynamic cutting tree algorithm to partition gene modules. (D) Modular-trait heatmap of correlation between gene modules and LMA. Each module contains the corresponding correlation coefficient and p-value.

Firstly, the hclust function was used to perform hierarchical clustering on 21,297 genes in 20 samples to eliminate outlier samples [24]. As shown in Fig 4A, 19 samples were retained for subsequent analysis. Second, the scale-free co-expression gene network was established with the one-step network construction function, and the soft threshold (*β*) was calculated using the R function “ pickSoftThreshold “ (Fig 4B). Then, the adjacency matrix was transformed into topological overlap matrix (TOM) and the corresponding dissimilarity (1-TOM) was calculated. Using hierarchical clustering and dynamic tree cutting to detect the modules in the network. Modules with a height value lower than 0.25 were merged, resulting in 19 distinct modules. Correlation analysis between these 19 modules and LMA traits revealed that the sienna3 and lightcyan1 modules were strongly positively correlated with LMA.

### Hub genes screening in the sienna3 module and lightcyan1 module

**Fig 5:**
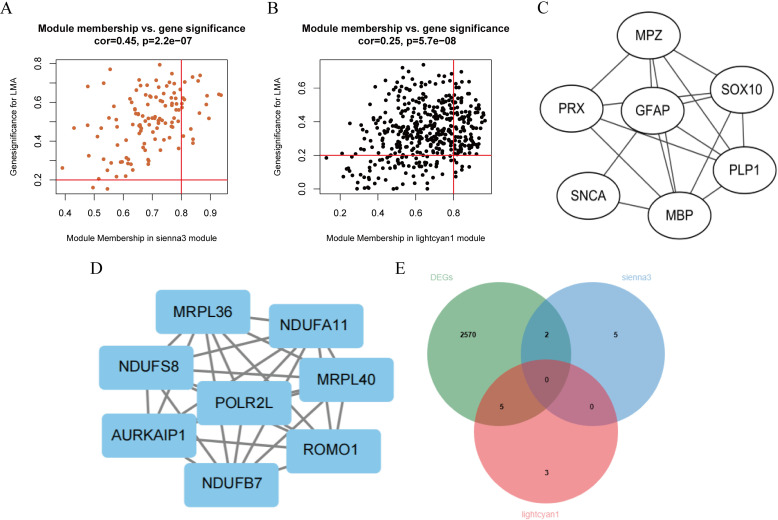
Intersection gene screening. (A) Hub gene within the sienna3 module. (B) Hub gene within the lightcyan1 module. (C) Intersection genes obtained by MCC, MNC, and Degree algorithms in the sienna3 module. (D) Intersection genes obtained by MCC, MNC, and Degree algorithms in the lightcyan1 module. (E) The intersection of genes selected by three algorithms and DEGs.

Hub genes in the sienna3 module and lightcyan1 module were defined with GS was greater than 0.2 and MM greater than 0.8. The results showed there were 18 Hub genes in the sienna3 module (Fig 5A) and 105 Hub genes in the lightcyan1 module (Fig 5B), respectively. Then, the top 10 genes of hub gene in lightcyan1 module and the top 10 genes of all genes in sienna3 module were selected according to MCC, MNC and Degree calculated by Cytohubba plug-in in Cytoscape software. At the same time, the intersection of genes obtained by the three algorithms was taken (Fig 5C and 5D). Subsequently, these initially selected genes were further intersected with DEGs and the overlapping genes including *PLP1*, *SNCA*, *MRPL36*, *NDUFS8*, *NDUFB7*, *NDUFA11*, and *MRPL40* were determined for further analyzed (Fig 5E).

## Discussion

LMA is the cross-sectional area of the longest dorsal muscle of livestock, which is particularly important in breeding because of its strong correlation with livestock meat production performance. In general, a larger LMA indicates a relatively higher pure meat percentage and better meat quality, while cattle with a larger LMA tend to have fuller and more developed muscles, which may also imply that they grow faster. Previous studies combined GWAS with RNA-seq to reveal that *HDAC9* is a candidate gene affecting LMA in Beijing black pigs [29]; Two quantitative trait loci (QTL) related to LMA were identified on SSC1 and SSC2 by multi-species genome-wide association analysis. The candidate gene for the QTL on SSC1 is *FUBP3* (far upstream element-binding protein 3) [30]. Zhao X et al [31] identified one (*DOCK5*), three (*PID1*, *PITX2*, *ELOVL6*), and three (*CCR1*, *PARP14*, *CASR*) genes as candidate genes for LMA, loin muscle depth (LMD), and BFT using GWAS.

In this study, four significant SNPs were screened at the genome-wide level and annotated to be located in or close to three coding genes. The *THBD* gene encodes Thrombomodulin, a protein primarily expressed on the surface of endothelial cells. The *THBD* gene is enriched in GO pathways related to calcium ion binding and blood coagulation.The *THBD* gene played vital roles in the muscle regulatory networks in the Yorkshire breed [32], which is consistent with the results of this study. *CD93*, also known as complement component C1q receptor, plays a key role in cell proliferation and migration [33]. The *CD93* gene exacerbated cell proliferation, angiogenesis, and immune evasion in osteosarcoma by triggering the PI3K/AKT pathway [34]. The *RIMS2* gene encodes the regulatory synaptic membrane exocytosis 2 protein and affects metabolic pathways related to cell differentiation and proliferation. It is also associated with the regulation of the immune system.

In this study, seven candidate genes were screened at the transcriptome level, with one overlapping gene with the GWAS results. Pathway enrichment analysis showed proteolipid protein l (*PLP1*) was mainly enriched in the signaling pathway associated with mental illness, and was also involved in the long-chain fatty acid biosynthetic process (GO:0042759). *PLP1* was the main component of myelin, and its mutation will lead to progressive neurodegeneration and eventually death due to severe protein defects [35], thus its dysfunction will directly affect the motor function of muscles. *PLP1* was also involved in the regulation of intestinal motility and barrier function [36]. The α-synuclein (*SNCA*) was involved in Parkinson’s disease (hsa05012), actin binding (GO:0003779) and other related pathways. *SNCA* may play a role in the compartmentalization of acetylcholine at the neuromuscular junction and in the fine control of skeletal muscle activity [37].

*MRPL36* and *MRPL40* both belong to mitochondrial ribosomal proteins, which are important components of the structural and functional integrity of the mitochondrial complex [38]. The overexpression of *MRPL36* seems to improve the efficiency of mitochondrial translation [39]. Ying Liu et al. found that *MRPL40* may be a potential target for the treatment of cryptorchidism and decreased sperm motility and number [40]. Moreover, the downregulation of *MRPL40* expression can eliminate the effect of interleukin-8 (IL-8) on promoting the proliferation and migration of rectal cancer [41]. Currently, there was no direct evidence to show that *MRPL36* and *MRPL40* were involved in the genetic regulation of muscle development. However, as key components of mitochondrial ribosomal proteins, their contribution to mitochondrial function and energy metabolism may indirectly affect muscle development and maintenance.

*NDUFS8*, *NDUFB7*, and *NDUFA11* were three genes related to the mitochondrial respiratory chain, and the proteins encoded by these three genes were all important components of the mitochondrial respiratory chain Complex I (NADH). All three genes were involved in biological processes such as citric acid (TCA) cycling respiratory electron transport (R-HSA-1428517) and respiratory electron transport (R-HSA-611105). The expression level of *NDUFS8* in myoblasts decreased significantly with the increase of age, and experimental results showed that the expression of *NDUFS8* in myoblasts promoted differentiation, self-renewal, and apoptosis resistance [42]. Knockout of *NDUFS8* inhibits cell proliferation, migration, and capillary formation in cultured endothelial cells [43]. *NDUFB7* and other genes were involved in regulating lipid synthesis and fatty acid *β*-oxidation, to maintain normal energy supply under low oxygen conditions [44]. In chickens with woody breast meat, the expression of the *NDUFB7* gene, which was associated with mitochondrial and ATP synthesis, tends to decrease. Additionally, other studies have shown that *NDUFA11* expression was significantly reduced in the blood of patients with acute myocardial infarction, making it a potential biomarker for diagnosis of myocardial infarction [45].

The genes *PPP3R1*, *FAM129B*, and *UBE2G1* are key genes influencing the LMA of uncastrated Nellore males [11]. These genes do not overlap with those identified in this study, which is likely due to differences in breeds.

## Conclusions

This study described the genomic variants and candidate genes associated with LMA in Huaxi cattle. Through genome-wide association analysis, the *THBD* gene and the *CD93* gene on BTA 13, and the *RIMS2* gene on BTA 14 were detected to be crucial in affecting the LMA trait. Transcriptome analysis showed that three other candidate genes (*PLP1*, *SNCA*, and *NDUFS8*) were also identified as candidate genes, which regulate LMA by participating in actin synthesis and muscle development. Summarily, the candidate genes related to LMA identified in this study were *THBD*, *CD93*, *RIMS2, PLP1*, *SNCA*, and *NDUFS8.* This result could provide new insights into the molecular mechanisms regulating LMA in beef cattle.

## Supporting information

S1 File **S1 Table.** Phenotype statistics of two groups of samples. **S2 Table.** The annotation of significant SNPs (±100 kb). **S3 Table.** The information of sequencing reads alignment to Bos taurus reference genome. **S4 Table.** The significant GO terms and KEGG pathways for differentially expressed genes. **S5 Table.** The enriched GO terms and KEGG pathways for the hub genes in two significant module. **S6 Table.** The detailed information of 6 genes associated with loin muscle area identified by GWAS and RNA-seq strategies.
(XLSX)
