# Correction: Integrating genomics and transcriptomics reveals candidate genes affecting loin muscle area in Huaxi cattle

**DOI:** 10.1371/journal.pone.0339330

**Published:** 2025-12-19

**Authors:** Qingqing Xue, Lili Du, Tianyu Deng, Mang Liang, Keanning Li, Li Qian, Shiyuan Qiu, Yan Chen, Xue Gao, Lingyang Xu, Zezhao Wang, Caihong Zheng, Lupei Zhang, Junya Li, Huijiang Gao

There are errors in the author affiliations. The correct affiliations are as follows:

Qingqing Xue^1,2^, Lili Du^2^, Tianyu Deng^2^, Mang Liang^2^, Keanning Li^2^, Li Qian^2^, Shiyuan Qiu^2^, Yan Chen^2^, Xue Gao^2^, Lingyang Xu^2^, Zezhao Wang^2^, Caihong Zheng^2^, Lupei Zhang^2^, Junya Li^2^, Huijiang Gao^2^

**1** College of Animal Science and Veterinary Medicine, Heilongjiang Bayi Agricultural University, Heilongjiang, China, **2** Institute of Animal Science, Chinese Academy of Agricultural Sciences, Beijing, China.
